# Case Report: Alzheimer's Dementia Associated With Cerebrospinal Fluid Neurochondrin Autoantibodies

**DOI:** 10.3389/fneur.2022.879009

**Published:** 2022-06-16

**Authors:** Niels Hansen, Berend Malchow, Bianca Teegen, Jens Wiltfang, Claudia Bartels

**Affiliations:** ^1^Department of Psychiatry and Psychotherapy, University Medical Center Goettingen, Göttingen, Germany; ^2^Euroimmun Laboratory, Lübeck, Germany; ^3^German Center for Neurodegenerative Diseases (DZNE), Göttingen, Germany; ^4^Department of Medical Sciences, Neurosciences and Signaling Group, Institute of Biomedicine (iBiMED), University of Aveiro, Aveiro, Portugal

**Keywords:** neurochondrin autoantibody, Alzheimer's disease, cerebrospinal fluid, autoimmunity, dementia

## Abstract

**Background:**

Neurochondrin autoimmunity is a rare disorder mainly associated with cerebellar and vestibular syndromes. Our report aims to enlarge its phenotypic spectrum to encompass major cognitive disorder with very late onset never before reported in conjunction with neurochondrin antibodies.

**Methods:**

We describe the case of an 85-year-old woman who presented in our memory clinic. Retrospective analysis of patient records included cerebrospinal fluid (CSF) analysis, magnetic resonance imaging (MRI), and neuropsychological testing using the CERAD-plus.

**Results:**

Because of her unknown onset of progressive cognitive dysfunction in conjunction with speech and language problems, we decided to take an extensive differential diagnostic approach including a search for neural autoantibodies potentially involved in cognitive impairment. Our patient presented serum and CSF neurochondrin autoantibodies. Further CSF analysis revealed elevated tau and ptau 181 protein as well as a reduced Aß42/40 ratio in CSF, thus matching a biomarker profile of Alzheimer's disease (AD). Neuropsychological tests revealed predominant and severe deficits in verbal and visual memory. Her MRI showed reduced parietal and cerebellar brain volume.

**Discussion:**

Taken together, this case reveals the novelty of a patient with a CSF-based and typical clinical and imaging profile of AD. She is also likely to have neurochondrin autoimmunity, as we detected neurochondrin autoantibodies in her CSF; we therefore diagnosed AD dementia associated with neurochondrin antibodies. Our case expands the spectrum of neurochondrin autoimmunity to disorders involving major cognitive disorder such as AD dementia. Furthermore, we speculate that neurochondrin autoimmunity might have triggered an acceleration of AD symptoms as its onset was reported only after a short 6-month interval via a synergistic or negatively additive hybrid mechanism of action between neurodegeneration and autoimmunity.

## Introduction

Neurochondrin is an intracellular, neuronal protein in the cytosol that is a target of antibodies associated with neuropsychiatric conditions such as cerebellar ataxia, brainstem signs, rhombencephalitis, psychosis (1), and hyperkinetic movements such as chorea (2). Other symptoms such as cognitive dysfunction including memory disturbances and behavioral symptoms have been reported in children as occurring in conjunction with neurochondrin autoantibodies (3). These reports are evidence of neurochondrin autoantibodies coinciding with cognitive dysfunction. However, to our knowledge, no patient with dementia and a typical CSF profile indicative of Alzheimer's disease (AD) has been shown to be associated with serum and CSF neurochondrin autoantibodies until now. Our case highlights neurochondrin IgG autoimmunity in a patient suffering from progressive AD dementia.

## Case Report

**Figure 1:**
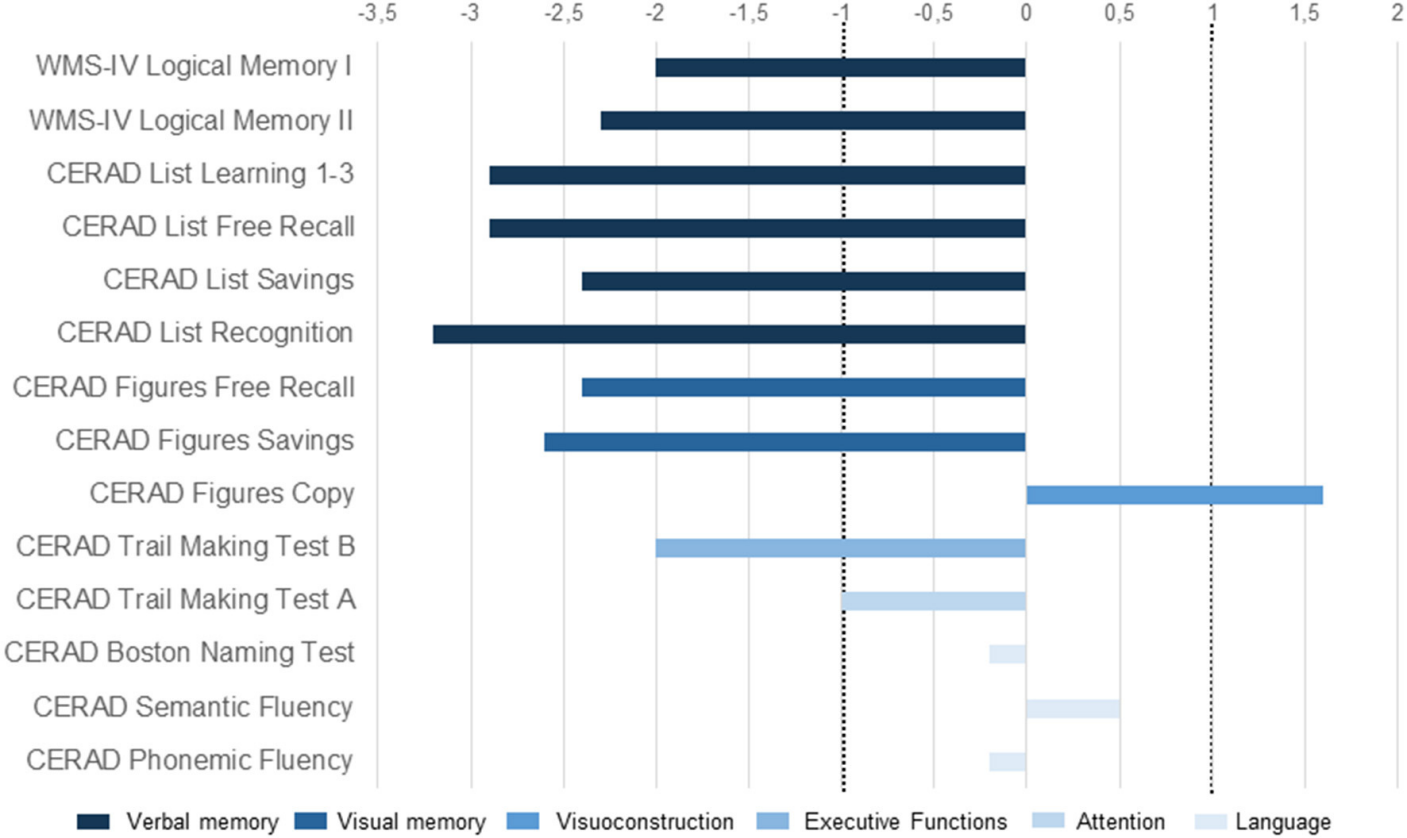
Cognitive profile (clinical case with neurochondrin antibodies in association with AD dementia). Illustration of neuropsychological test results presented as z-scores. The area between dotted lines denotes the normal range. CERAD, Consortium to Establish a Registry for Alzheimer's Disease; WMS-IV, Wechsler Memory Scale–Fourth Edition.

An 85-year-old woman, retired and living on her own, presented in our memory clinic complaining of a progressive loss of spatial and time orientation and memory probably for 6 month. Her son, who is also her guardian, also mentioned slight speech and language problems, noticing disrupted sentences in her spontaneous speech, and difficulty speaking caused by word-finding problems; she was also impaired in performing everyday tasks and showed signs of social squalidness. Our patient has two sons. She lived first with one of them in another city. However, as he had to look after for another needy member of his family, he had no time to take care of his mother. Her condition worsened slowly and finally she lived in squalid conditions, so that her other son (and actual guardian) took her to his home in another city where he looked after her. It was there that he noticed her worsening memory and language problems. Both sons have been reporting memory disturbances for about 6 months. However, when exactly her symptoms' first onset appeared is unknown, thus a slowly progressive and no abrupt decline is most probable. This patient had no prior mental health or neurological disorder. She had several comorbidities that were treated pharmacologically as indicated in the following in brackets: (1) diabetes mellitus type 2 (metformin 2000 mg/d, glimepirid mg/d), (2) arterial hypertension (olemsartan /amlodipine/ hydrochlorothiazide 40/ 5/ 12.5 mg/d, bisoprolol 10 mg /d), (3) dermatitis (methylprednisolone ointment two times daily), (4) paroxysmal atrial fibrillation (Apixaban 10 mg/d, digitoxin 0.07 mg/d, bisoprolol 10mg/d), (5) hypothyreosis (L-Thyroxin 50 μg/ d), (6) tinea pedis (ciclopirox crème), and a (7) presbyacusis. The patient underwent a comprehensive dementia assessment. Her psychopathological assessment confirmed deficits in orientation, concentration, and memory. Furthermore, she had a reduced awareness of her cognitive abilities. In her neurological examination, no neurological deficits were detected. Cognitive screening results were normal for the clock drawing test (CDT = 2), but cognitive impairment became already obvious in the Mini-Mental Status Test (MMST = 21). Neuropsychological assessment further revealed impairments in cognitive flexibility and severe deficits in verbal and visual memory, including immediate and delayed recall (Figure 1). Together with impaired activities of daily living, these findings were compatible with a diagnosis of mild dementia. MRI showed reduced parietal and cerebellar brain volume and microangiopathy, as well as a central necrosis in the corpora mammillaria. The unknown onset of symptoms with cognitive dysfunction accompanied by speech and language problems prompted us to perform an extensive differential diagnosis including a search for a wide spectrum of neural autoantibodies that could potentially be involved in the differential diagnosis of cognitive impairment such as neurochondrin autoantibodies. Neurochondrin autoantibodies have been reported to be associated with an encephalopathy and cognitive dysfunction (3). CSF analysis revealed neurochondrin autoantibodies in immunofluorescence testing (1:32). Furthermore, titin antibodies were detected in CSF via Euroline immunoblots. Both autoantibody types [neurochondrin (1:3,200) and titin] were also verified in blood samples. We found no specific intrathecal antibody synthesis of anti-neurochondrin antibodies in the central nervous system with an antibody specific index of 2.85 [cut off is higher than 4 for indirect immunohistochemistry due to non-linear quantification according to Reiber and Peter (4), Table 1]. Furthermore, we did not detect isolated oligoclonal bands in the CSF or an intrathecal IgG synthesis. However, a. Neurodegenerative markers were above normative levels in CSF (measured in the Neurochemistry Cerebrospinal Fluid Laboratory of the Neurology Department, University Medical Center Göttingen), i.e., tau protein and ptau 181 protein (Table 1) were above normative levels, and the ratio Aß42/40 was below normative levels (Table 1). Results of neurodegenerative CSF markers were confirmed by an external laboratory (Laboratory of Clinical Neurochemistry and Neurochemical Dementia Diagnostics, Department of Psychiatry and Psychotherapy, University Hospital Erlangen) (Table 1, second column). To assess brain damage, neurofilament phosphorylated heavy chain (pNfH) were determined in CSF at the Department of Neurology, University Hospital Ulm (Table 1). However, laboratory reference values adapted from the work of Steinacker et al. (5) from a cohort with amyotrophic lateral sclerosis indicated no levels above normative levels (Table 1). AD dementia is probable due to her typical hippocampal subtype of memory dysfunction together with biomarker-based AD (elevated ptau181, reduced Aß42/Aß40 ratio). Our patient ‘s normal Aß42 in the CSF fails to support the hypothesis of Alzheimer's disease. Nevertheless, the combination of a reduced Aß42/40 ratio and elevated ptau181 in the CSF argues for a biomarker-confirmed Alzheimer's disease concurring with the latest diagnostic criteria (6). However, as the coexistence of neurochondrin autoantibodies in her serum and CSF are relevant in our patient, we thus diagnosed an AD dementia associated with neurochondrin autoantibodies. A therapy with cholinesterase inhibitors rivastigmine at 4.6 mg per day has been started and is well tolerated so far. No adverse events were reported. 40 mg/d pipamperone was also applied to help her sleep. Immunotherapy with corticosteroids as an individual off label therapy was briefly discussed, but not considered as a therapeutic option, as AD is very likely caused by a biomarker profile and typical clinical syndrome entailing memory and orientation disturbances. The patient and her son accepted the diagnosis of AD with dignity. Potential neurochondrin autoimmunity was not mentioned as a separate diagnosis as it is not currently a diagnostic category for such a clinical presentation.

**Table 1:** Clinical and laboratory parameters.

Demographic parameters	
Age y	85
Onset y	85
Psychopathology	
Disturbances of orientation	+
Disturbances of attention and memory	+
Formal thought disorders	−
Disturbances of affect	−
Disorders of drive and psychomotor activity	−
CSF	
Cell count (<5μg/L)	1
Lymphoctes in %	91
Monocytes in %	9
Lactat (mmol/l)	2.7
Total protein content (mg/L)	550
Albumin mg/L	318
IgG mg/L	29.1
IgA mg/ L	2.8
IgM mg/ L	1.6
Quotient Albumin %	8.1
Quotient IgG %	3.5
Quotient IgA %	1.7
Quotient IgM %	0.68
Neurochondrin Ab	1:32 ++
Titin Ab	++
Measles-AI	1.2
Rubella-AI	1.1
VZV-AI	1.1
HSV-AI	1.2
Tau protein (1: <450pg/ml, 2: <320 pg/ml)	1:1056, 2:1111
P Tau protein 181 (1: <61pg/ml, 2: <50 pg/ml)	1:198, 2:133.4
Aß42 (1:>450pg/ml, 2:>620pg/ml)	1:674, 2:861
Aß40	16850
Ratio Aß42/40 (1: x10: >0.5, 2: >0.05)	1:0.4, 2:0.04
pNfH (<560 pg/ml)	526
Intrathecal IgG synthesis	−
Isolated CSF oligoclonal bands	−
Antibody specific index (ASI) (<4)	2.85
Blood	
Albumin g/L	39.2
IgG g/L	8.3
IgA g/L	1.6
IgM g/L	2.3
Neurochondrin Ab	1:3200 +++
Titin Ab	++
CRP mg/l (≤ 5 mg/l)	3.0
Procalcitonin (≤ 0.07μg/L)	0.03
B-Leukocytes 10^∧^ 3μl	6.09

*Aß42, ß-amyloid 42; Aß40, ß-amyloid 40; CSF, cerebrospinal fluid; CRP, C reactive protein; IgA, immunoglobulin A; IgG, immunoglobulinG; IgM, immunoglobulin M; MRI, magnetic resonance imaging; P Tau Protein 181, phosphorylated tau protein 181; pNfH, neurofilament phosphorylated heavy chain; ratio Aß42/40, ratio ß-amyloid 42/ ß-amyloid 40; y, years; μg, microgram; g, gram; L, liter; mg, milligram; pg, picogram*.

*1: reference values and data from the Neurochemistry Cerebrospinal Fluid Laboratory of the Neurology Department, University Medical Center Göttingen. 2: reference values and data from the Laboratory of Clinical Neurochemistry and Neurochemical Dementia Diagnostics, Department of Psychiatry and Psychotherapy, University Hospital Erlangen. +, present; −, not present*.

## Discussion

Our novel finding of neurochondrin autoantibodies in conjunction with AD dementia seems surprising at first glance, as neurochondrin autoimmunity has only been reported so far in association with vestibulocerebellar syndromes (1, 7–9) and movement disorders (2, 10) and only rarely with cognitive dysfunction in children (3). Neurochondrin is required during embryogenesis, as embryos lacking the neurochondrin gene die early during their embryogenesis (11). Furthermore, neurochondrin is involved in neuritic outgrowth and spatial learning processes (12)—evidence of the potential role of neurochondrin autoimmunity in cognitive processes such as in our patient. However, as neurochondrin is an intracellular protein, the autoantibodies are not believed to be pathogenic themselves; additional T-cell mechanisms might be the main pathomechanism of brain damage in our patient. More investigations employing flow cell cytometry should explore whether activated CD8+ T-cells can be detected as an indirect sign of an immune mechanism in such patients. It remains unclear but more likely that autoantibodies would develop in a patient with existing AD pathology, although it is also conceivable that an early manifestation of neurochondrin autoimmunity might trigger neurodegenerative processes leading to an AD pathology. Neurochondrin is located in structures also affected in AD, such as the hippocampus, indicating that neurodegenerative processes might be accelerated by neurochondrin autoantibodies, as they also target synaptic structures within the hippocampus, as shown by Shelly et al. (1). Neurochondrin IgG is often found in the hippocampus and in its subregions (like CA2 and CA3) as well as the dentate gyrus (1). As the hippocampus is the pivotal structure for forming memories, it is not surprising that our patient has memory dysfunction given the neurochondrin IgG deposition in her hippocampus. Furthermore, it is important to mention that neurochondrin (also known as norbin) interacts with glutamatergic signaling, as it interacts with mGluR5 receptors in mice (13). If we transfer such findings from mice to man, the interaction of neurochondrin IgG with mGluR5 receptors in the hippocampus would result in memory dysfunction caused by disrupted synaptic transmission within the hippocampus. We believe that this postulated mechanism may underly our patient's progressive severe memory deficits. From another perspective, the neurodegenerative and autoimmune mechanisms of action might not interact, but they might have a negative and synergistic impact on cognitive function. Neurochondrin autoimmunity might ultimately have accelerated the clinical manifestation of AD symptoms, or have led even to the appearance of clinical symptoms. Nevertheless, it has to be emphasized that the occurrence of neurochondrin autoantibodies in a patient with typical AD is surprising, in particular as neurochondrin autoantibodies mostly have been reported up to now with other clinical features such as cerebellar ataxia (1, 9) or movement disorder (2). However, as proof of CSF neurochondrin autoantibodies accompanying clinical features of an intermittent anomic aphasia imply at least a coexisting autoimmune origin according to recent criteria (14), the neurochondrin autoantibodies are probably not merely incidental, despite clinical symptoms somewhat resembling those typical of AD. Nevertheless, we emphasize that the pathological significance of neurochondrin autoantibodies in the context of dementia and Alzheimer's disease is unclear because of the shortage of data. Furthermore, as no specific actual autoantibody-production in the CSF (such as an intrathecal IgG synthesis) was detected, neurochondrin autoimmunity's pathological significance is limited. However, as we had no access to left over biomaterial probes, no additional calculations were possible that might have highlighted the pathological significance of autoantibodies, as the determination of an elevated specific anti-neurochondrin antibody index would have done.

Taken together, our report demonstrates neurochondrin autoimmunity in a patient with an AD dementia, thereby expanding the extent of neurochondrin autoimmunity spectrum reported so far. The prognosis if neurochondrin autoimmunity at present is unclear, and no study data exists. The onset of AD might be accelerated by neurochondrin autoimmunity although the data is limited. Nevertheless, as there is no follow-up data on this patient, her prognosis over time course cannot be evaluated. Further research on neurochondrin autoimmunity in AD patients has considerable potential regarding the interaction between neurodegeneration and neural autoantibody-mediated immunity. The theoretical co-existence of neurochondrin autoantibodies in AD pathology might lead to two possible scenarios if a pathological significance of anti-neurochondrin autoantibodies is assumed: (1) worsening symptoms due to slowly progressing AD that is exacerbated by additional clinical features of neurochondrin autoimmunity and (2) the remission and relapse of some clinical features might reflect a potential time course of an autoantibody-related CNS disease. Because of the lack of data so far, it is unclear which condition our patient will suffer. Further studies will have to clarify whether neurochondrin antibodies themselves have pathogenic relevance for cognitive dysfunction, or if they co-exist with other neurological diseases such as ataxia or myelopathy (15).

## Data Availability Statement

The raw data supporting the conclusions of this article will be made available by the corresponding author, without undue reservation.

## Ethics Statement

Ethical review and approval was not required for the retrospective case report on a human participant in accordance with the local legislation and institutional requirements. The patient provided written informed consent for publication of her data.

## Author Contributions

NH wrote the manuscript. BM, BT, JW, and CB revised the manuscript for important intellectual content. All authors contributed to the article and approved the submitted version.

## Funding

Funding was received from the Open access fund of the University of Göttingen. JW was supported by an I.P. iBiMED (UIDB/04501/2020) at the University of Aveiro, Portugal.

## Conflict of Interest

The authors declare that the research was conducted in the absence of any commercial or financial relationships that could be construed as a potential conflict of interest.

## Publisher's Note

All claims expressed in this article are solely those of the authors and do not necessarily represent those of their affiliated organizations, or those of the publisher, the editors and the reviewers. Any product that may be evaluated in this article, or claim that may be made by its manufacturer, is not guaranteed or endorsed by the publisher.
